# Improving the Sensory Properties of Layered Phospholipid-Graphene Films Due to the Curvature of Graphene Layers

**DOI:** 10.3390/polym12081710

**Published:** 2020-07-30

**Authors:** Michael M. Slepchenkov, Olga E. Glukhova

**Affiliations:** 1 Department of Physics, Saratov State University, Astrakhanskaya Street 83, 410012 Saratov, Russia; slepchenkovm@mail.ru; 2 Laboratory of Biomedical Nanotechnology, I.M. Sechenov First Moscow State Medical University, Bolshaya Pirogovskaya Street 2-4, 119991 Moscow, Russia

**Keywords:** sensory properties, phospholipid–graphene films, curvature, charge transfer, electrical conductivity, density-functional tight-binding method, electron charge density, current-voltage characteristic

## Abstract

This article is devoted to the in silico study of the sensory properties of mono- and bilayer phospholipid-graphene films with planar and curved graphene sheets. The DPPC (dipalmitoylphosphatidylcholine) molecules are considered as phospholipid structures. These molecules are part of lipid bilayers, liposomes and cell membranes. To find a way to improve the sensory properties of phospholipid-graphene films, we studied the effect of the curvature of the graphene sheet on the charge transfer and electrical conductivity of the films. The distribution of the electron charge density over the film atoms was calculated using the self-consistent-charge density-functional tight-binding method (SCC-DFTB). The calculation of the current through phospholipid-graphene films was carried out within the framework of the Landauer–Buttiker formalism using the Keldysh nonequilibrium Green function technique. As a result of the calculations, the optimal configuration of the arrangement of DPPC molecules between two graphene layers was established. This configuration provides the maximum possible increase in current to 1 μA at low voltages of ~0.2 V and is achieved for curved graphene with a radius of curvature of ~2.7 nm at individual points of graphene atomic network.

## 1. Introduction

Graphene-based materials have attracted increasing attention due to their two-dimensional structure of atomic thickness, large specific surface areas and excellent electro-conductive and mechanical properties [1]. The combination of graphene-based materials with biomolecules offers a promising method for creating new hybrid graphene-biomolecule nanomaterials with unique functions in medicine, biology, nanotechnology and materials science [2,3,4]. One of these hybrid nanomaterials is the graphene-phospholipid biomolecular complex [5,6,7,8]. The impetus for the study of the graphene-phospholipid complexes was given after the publication of a number of scientific papers that described the technology of experimental production of layered graphene by exfoliation of graphite in liquid colloidal solutions in the presence of phospholipids. In particular, Pykal et al. developed a one-step method for exfoliating graphene in a lecithin/chloroform solution [9]. Using molecular dynamic (MD) modeling and spectroscopy methods, scientists have shown that the effectiveness of graphene exfoliation can be significantly improved by adding lipids, namely lecithin. A. T. Williams et al. developed a simple, inexpensive and “green” technology for production of graphene in colloidal dispersion [10]. The one-stage method proposed by the authors makes it possible to obtain aqueous dispersions of phospholipid-coated graphene by ultrasonic exfoliation of graphite in water.

Graphene-phospholipid structures are comprehensively studied using both experimental research methods and computer simulation methods [11,12,13,14,15,16,17]. Using computer modeling methods, the interaction between lipid membranes and graphene is studied in detail. In particular, Moore et al. carried out MD simulation of the interaction between lipid membranes and graphene nanoscales coated with single-stranded DNA using the CHARMM force field [16]. It was demonstrated that a partial coating of single-stranded DNA reduces the penetration depth of the graphene nanoflakes into the phospholipid bilayer by weakening the hydrophobic force that controls the penetration. The authors concluded that a DNA coating can reduce the graphene cytotoxicity by shielding the adverse graphene-water interaction, thus preventing the penetration of graphene and lipid extraction. Dallavalle et al. performed coarse-grained MD simulation of the interaction of graphene oxide (GO) sheets of various structures with a phospholipid bilayer [17]. During the simulation, they found that GO sheets can penetrate through the phospholipid membrane and move along the bilayer only in a case of random distribution of hydrophilic groups in the structure. If the oxidation is located out at the sheet edges, GO sheet hover over the membrane and causes a serious reorganization of lipids.

The use of graphene-phospholipid complex is very promising in a wide range of biological applications [18,19,20,21,22,23,24,25,26,27,28]. For example, phospholipid molecules coating graphene can enhance the antibacterial effect of laser-induced graphene [19]. Graphene-phospholipid structures have great potential for use as material interfaces for devices and biomedical tools designed to modulate or restore brain functions. For example, Durso et al. proposed a new strategy based on the chemical modification of GO with synthetic phospholipid (PL) to improve the interaction of GO with astroglial brain cells [20]. The researchers showed that astrocytes seeded on the GO-PL hybrid structure did not show significant gliotic reactivity, indicating that the material interface did not cause a harmful inflammatory reaction when interacting with astroglial cells. Due to the high mobility of charge carriers and semimetal conductivity, graphene is a promising material for use as a substrate in the assembly of lipid membranes used in various fields of bioengineering [21,22,23,24,25]. Thus, Hirtz et al. demonstrated the assembly of active lipid membranes on graphene of a controlled location and size with submicron resolution [22]. It was shown that the multiplex assembly of phospholipid membranes of different functionality in close proximity to each other can be achieved on graphene using Dip-pen nanolithography (L-DPN) technology. The use of L-DPN technologies allows critical control over the chemical composition and spatial distribution lacking in self-organizing membranes. Willems et al. investigated the molecular properties of supported lipid membranes on graphene and graphene oxide substrates using atomic force microscopy and coarse-grained MD simulations [24]. It was demonstrated that lipids can form multilayer structures such as 1.5 bilayers on the hydrophilic surfaces of GO and can spontaneously rearrange, forming a preferred topology regardless of the starting structures (e.g., regular bilayers). It was established that the hydrophobic surface of pure graphene interacts very favorably with lipids, affecting the stability of the lipid bilayer membranes on this surface. One of the most promising areas of application of graphene–lipid structures is biosensorics. Various biosensor devices have been developed based on lipid-modified graphene [26,27,28]. The urgent task in the framework of the development of this field is to improve the sensitivity and stability of the lipid/graphene system-based sensors.

The objects of this study are mono- and bilayer phospholipid-graphene films. The monolayer film is formed by a graphene sheet with adsorbed DPPC molecules. The bilayer film consists of two graphene monolayers with DPPC molecules between them. The aim of this paper is to find a way to improve the sensory properties of phospholipid-graphene films by curving graphene monolayers using in silico computational methods. In this paper, we consider only DPPC (dipalmitoylphosphatidylcholine) molecules, since they are part of the cell membrane and liposomes used actively in biomedicine [29,30,31,32,33], although other types of phospholipid molecules can also be used.

## 2. Computational Details

The search for the equilibrium configuration of the monolayer film supercells was carried out using the SCC-DFTB method [34], which has proven itself very well in studies of the interaction of carbon nanostructures with various biomolecular objects [35]. The SCC-DFTB method allows one to study the electronic structure, energy and density of electronic states distribution for nanomaterials with supercells containing several hundred atoms and even thousands of atoms, which cannot be realized using ab initio methods. This method is implemented in the DFTB+ (Bremen Center for Computational Materials Science, University of Bremen, Bremen, Germany) [36] and Kvazar–Mizar packages (Department of Physics, Saratov State University, Saratov, Russia) [37,38], which are used in this paper. To identify the equilibrium configuration of the bilayer film supercells, a MD study was carried out using the SCC-DFTB method to refine the energy value. The MD study allows one to track the behavior of a DPPC molecule in the presence of two graphene layers, as well as to establish its equilibrium position and structure. The time step of MD simulation was 0.1 fs. The distribution of the electron charge density over the film atoms was calculated using the SCC-DFTB method. An analysis of the atomic populations was carried out according to the Mulliken procedure [39].

Calculation of the current through phospholipid-graphene film was carried out within the framework of the Landauer–Buttiker formalism [40] according to the formula:

(1) $$I=\frac{e}{\hbar }\int_{-\infty }^{\infty }T\left(E\right)dE\left[f_{1}\left(E\right)-f_{2}\left(E\right)\right]$$

where *T*(*E*) is the transmission function, which determines the total quantum-mechanical transparency of the conductive structure over all independent conduction channels for an electron with energy *E*. *f*_1_ and *f*_2_ are the Fermi–Dirac functions that characterize the energy levels of the source (electrode 1) and drain (electrode 2), corresponding to the energy level of the conducting structure. The electron transmission function *T*(*E*) and current *I* were calculated using the Mizar program (Department of Physics, Saratov State University, Saratov, Russia) [38]. This program implements the Landauer–Buttiker approach and the non-equilibrium Green’s function (NEGF) formalism [41] for studying the quantum electron transport during current transfer in nanoscale structures taking into account elastic electron scattering by atomic network inhomogeneities. In our case, the supercell of the phospholipid-graphene film contains 336–616 atoms of graphene layers participating in the current transfer. This requires the application of the method for accelerating the calculation of *T*(*E*), otherwise the computational resources are insufficient to calculate the transmission function using the SCC-DFTB method. In this study, to speed up the calculation of *T*(*E*) of polyatomic supercells, we used the original method implemented in the Mizar program software [42]. This method allows one to calculate the *T*(*E*) for a small number of k-points of the first Brillouin zone and then interpolate it for any k-point of the first Brillouin zone and obtain the complete transmission function *T(E)*.

## 3. Results and Discussion

### 3.1. Atomistic Models and Electron Properties of Monolayer Phospholipid-Graphene Films

**Figure 1:**
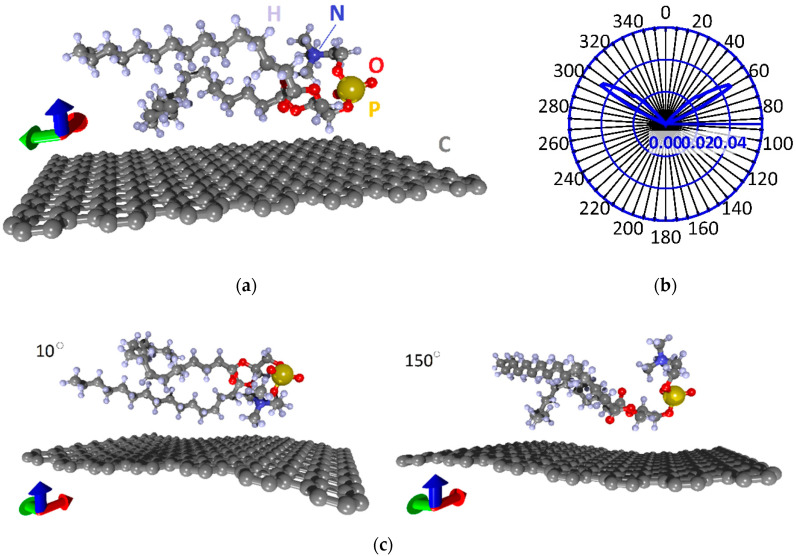
Atomistic models of the supercell of a monolayer phospholipid-graphene film: (**a**) initial atomic configuration of the supercell; (**b**) distribution of ΔE during the rotation of the DPPC molecule; and (**c**) equilibrium configurations of the supercell at rotation angles of 10 and 150°. Colors of the axes (hereinafter): red, *x*-axis; green, *y*-axis; blue. *z*-axis.

To study the properties of phospholipid-graphene films, first, it is necessary to construct energetically favorable (equilibrium) atomistic models of the film supercells. Therefore, we first carried out a search for the equilibrium configuration of the arrangement of the DPPC molecule on the graphene monolayer. Figure 1a shows the initial supercell of a monolayer phospholipid-graphene film. One supercell contains a graphene monolayer (252 atoms) and one DPPC molecule (130 atoms), the head of which is formed by one phosphorus atom (P), one nitrogen atom (N), seven oxygen atoms (O) and also carbon (C) and hydrogen (H) atoms. Two tails of DPPC are formed by C–H chains. The X × Y dimensions of the supercell are 22.14 × 29.82 Å, respectively. Such a supercell size corresponds to the smallest distance between DPPC molecules that do not interact with each other (as if isolated from each other). The smallest distance between the DPPC molecule and the graphene layer was ~3 Å.

To identify the energetically favorable DPPC configuration relative to the graphene monolayer, the relaxation scanning approach was implemented. It consists in sequentially rotating the DPPC molecule around its axis (passing through the center of mass and parallel to the *y*-axis) by one degree, while optimizing the atomic structure of the entire supercell. Double optimization was carried out when all the coordinates of all atoms and the translation vectors of the supercell along the X and Y directions act as variable parameters. The optimization criterion was the value of the total energy *E_tot_*. That is, the supercell structure was considered as optimized when a global minimum of total energy was achieved for this atomic configuration. Thus, 360 optimization procedures for the DPPC-graphene structure were carried out.

Figure 1b shows the polar (radar) chart of the relative deviation of $E_{tot}$ from its minimum value $E_{tot}^{min}$ during successive rotation. This value Δ*E* was calculated as follows:

(2) $$\Delta E=\frac{E_{tot}-E_{tot}^{min}}{E_{tot}^{min}}\times 100\%$$

Taking into account the number of atoms in the supercell, the minimum value of energy $E_{tot}^{min}$ is −40.0494 eV/atom. That is, Δ*E* values close to zero correspond to the equilibrium configuration of the DPPC molecule relative to graphene. The polar chart shows that there are two localized groups of the DPPC molecule positions, which are characterized by maximum energy values. These polar lobes of the chart with the highest value of Δ*E* correspond to rotation angles of 60 ± 5°and 300 ± 5°. The rotation angle ranges 0–40, 75–295 and 320–360° correspond to the lowest values of energy *E_tot_* = −40.0494 ± 0.0002 eV/atom. Figure 1c shows the atomic configurations of the supercell of a monolayer phospholipid-graphene film with a minimum energy for rotation angles of 10 and 150°.

**Figure 2:**
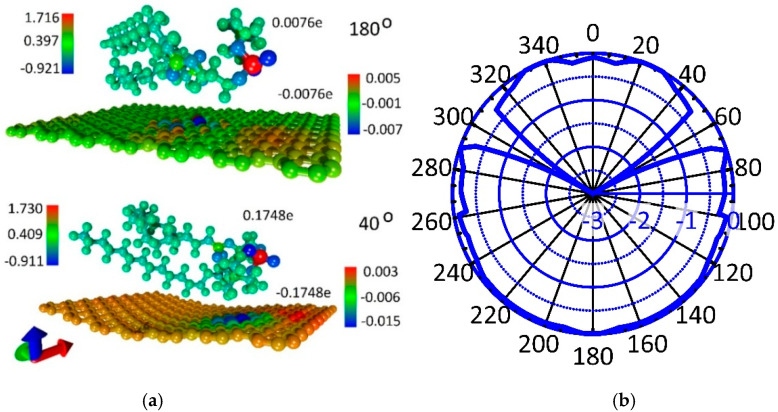
Charge transfer in a monolayer phospholipid-graphene film in fractions of electron charge *e*: (**a**) color illustration of the electron charge density distribution for the case of planar and curved graphene; and (**b**) the transferred charge on graphene from the DPPC molecule during its rotation.

Note that, in most cases of equilibrium supercell configurations, graphene takes a curved shape to varying degrees, i.e., adapts to the shape of the DPPC molecule. The interaction energy between the DPPC molecule and graphene is about −0.253 ± 0.051 eV, i.e., taking into account the graphene sheet size, the adhesion energy is ~(−0.038) ± 0.012 eV/nm^2^. A negative value of the energy indicates the exothermic process of the formation of this phospholipid-graphene complex. The electron density distributions and charge transfer between the DPPC molecule and the graphene sheet were calculated for all equilibrium configurations of the supercell of a monolayer phospholipid-graphene film. It was found that the supercell configurations with planar and curvilinear graphene differ very noticeably in value of the transferred charge. For comparison, Figure 2a shows color illustrations of the electron density distribution for the cases of planar (when turning through an angle of 180°) and curved graphene (when turning through an angle of 400°). The color scale shows the charge value in fractions of the absolute value of the electron charge *e*. The total charge values are indicated next to the DPPC molecule and graphene. It is clearly seen that the charge transfers from the DPPC molecule to graphene in both cases, while the DPPC-graphene complex itself remains neutral. The difference between the planar and curved graphene is that the charge transfer is 23 times greater in the case of curved graphene. The adhesion energy is also 15% higher in the case of curved graphene. The results of calculating the charge transfer from the DPPC molecule to graphene are presented in Figure 2b in the form of a polar (radar) chart displaying the charge in fractions of *e*. As expected, this chart is consistent with the polar chart in Figure 1b. The maximum of the transferred charge (−2e to −3e) corresponds to the maximum value of ΔE, when the DPPC molecule is located too close above the graphene surface, which makes this configuration non-equilibrium. For cases with a minimum energy ΔE, the charge on graphene is −0.16 ± 0.015e. It can be preliminarily concluded that, as a result of charge transfer, the electrical conductivity of graphene will increase, which will improve its sensory abilities. The graphene curvature is in the range 0–0.1737 nm^−1^, and the minimum radius of curvature is 5.757 nm.

### 3.2. Atomistic Models and Electron Properties of Bilayer Phospholipid-Graphene Films

**Figure 3:**
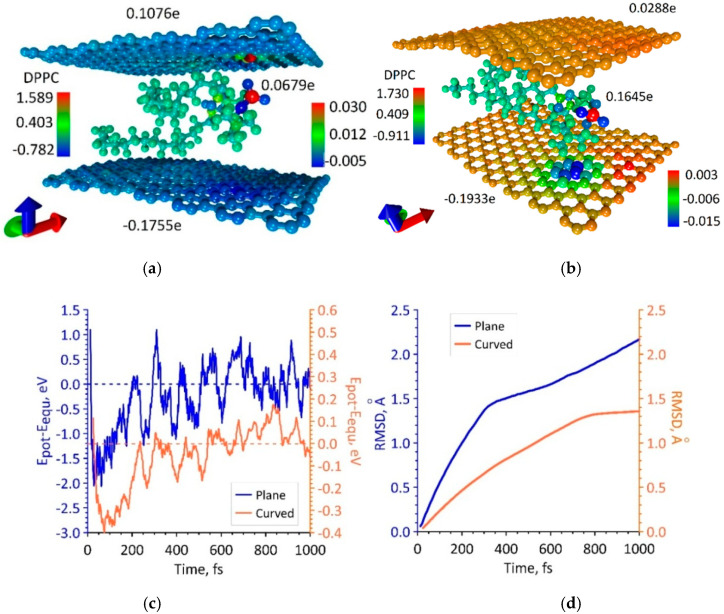
Atomistic models of the supercell of a bilayer phospholipid-graphene film: (**a**) configuration with planar graphene layers; (**b**) configuration with curved graphene layers; (**c**) change in the potential energy *E_pot_* relative to the energy of the film in the equilibrium state *E_equ_* during the search for the equilibrium configuration of the film supercell; and (**d**) RMSD graphs of DPPC atoms during the MD simulation.

Next, we determined the equilibrium configurations of the supercells of bilayer phospholipid-graphene films. The initial configuration was the equilibrium configuration of the supercells of monolayer phospholipid-graphene films obtained in the previous step. Supercells with planar and curved graphene sheets were considered. In the new supercells, one more graphene monolayer was added from the opposite side of the DPPC molecule. As in the case of a monolayer film, several configurations of a bilayer phospholipid-graphene film are equilibrium, both with planar graphene and with curvilinear. Two of these configurations are shown in Figure 3a,b. Figure 3c shows graphs of potential energy changes during MD modeling: the blue curve corresponds to the atomistic model of the bilayer phospholipid-graphene film presented in Figure 3a and the orange curve corresponds to the atomistic model presented in Figure 3b. These graphs show a change in the magnitude which is the difference between the potential energy *E_pot_* and the energy *E_equ_* corresponding to the equilibrium structure. The value of *E_equ_* was determined as the average value, around which the value of *E_pot_* fluctuates after a few picoseconds of MD simulation. The equilibrium energy *E_equ_* is −27,073.186 eV in the case of planar graphene sheet and *E_equ_* = −27,078.693 eV in the case of curved graphene sheet. In the case of planar graphene, upon reaching the equilibrium configuration of a phospholipid-graphene bilayer film, the *E_pot_* energy fluctuations amounts to an average of 0.357 eV; the maximum deviation from *E_equ_* value is not more than 0.01% (blue curve in Figure 3c). In the case of curved graphene, the *E_pot_* energy fluctuations near the equilibrium value have an even smaller amplitude of ~0.06 eV (0.0002%, orange curve in Figure 3c). The root mean square deviation (RMSD) graphs of DPPC atoms during the first picosecond of MD simulation are shown in Figure 3d. These graphs clearly show the difference between two supercell configurations. For a supercell configuration with curved graphene, the RMSD curve quickly reaches a plateau, which indicates the rapid achievement of a stable state of the DPPC molecule structure. The equilibrium configuration of the supercell of a bilayer graphene-phospholipid film with planar graphene is achieved only after 2 ps.

The large difference in the electronic structure of atomistic models shown in Figure 3a,b is also noticeable. In the first case (Figure 3a), both planar graphene sheets carry a charge of more than 0.1|*e*|; one graphene sheet is negatively charged, and the other graphene sheet is positive. A similar situation is observed for the second case with curved graphene sheets (Figure 3b); however, the pattern of the charge distribution is noticeably different. One can say that there is a large negative charge ~(−0.2e) on the bottom graphene sheet and practically no charge on the upper graphene sheet. A similar situation is observed for all atomistic models of a bilayer phospholipid-graphene film with curved graphene sheets: one sheet has a large negative charge, and the other sheet is practically not charged. The distribution of electron charge density on the lower sheet is strongly inhomogeneous, as shown in Figure 3b. In general, the pattern of the charge redistribution for these atomistic models of a bilayer graphene-phospholipid film allows us to conclude that the charge is taken by the DPPC molecule from the upper graphene sheet and transferred together with its charge to the bottom graphene sheet.

### 3.3. Phospholipid-Graphene Bilayer Films with Curved Graphene Providing Close Packing of DPPC Molecules and Charge Transfer

Based on the results presented in Section 3.1 and Section 3.2, we can conclude that the curved graphene most effectively interacts with DPPC molecules. In this regard, we further consider only atomistic models of phospholipid-graphene bilayer films with curved graphene and study ways for densification of DPPC molecules. The close packing of DPPC molecules will provide the greatest charge transfer to graphene and thereby increase its electrical conductivity and, as a result, improve its sensory properties. We considered two ways for increasing the packing density. One way is to narrow the original supercell in the direction of *x*-axis, the other way is to place two phospholipid molecules in one supercell at once. To identify the most effective way, the comparative calculations have been performed.

**Figure 4:**
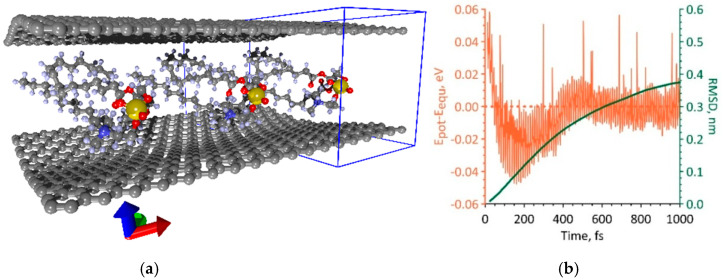
The first variant of dense packing of DPPC molecules in a bilayer phospholipid-graphene film: (**a**) atomistic model of a bilayer film; and (**b**) changes in the potential energy *E_pot_* relative to energy of the film in the equilibrium state *E_equ_* and RMSD during the search for the equilibrium film configuration.

During the simulation of the first way of increasing the packing density of DPPC molecules, it was shown that a supercell of a bilayer phospholipid-graphene film with dimensions of 14.76 × 29.81 Å is energetically favorable. One graphene layer in this supercell contains 168 atoms. The maximum packing density of DPPC molecules is achieved at such supercell sizes. Moreover, the DPPC molecules are strictly periodically located between the graphene layers and weakly interact with each other. Figure 4a shows a fragment of a similar phospholipid-graphene bilayer film including three supercells (the supercell is highlighted in blue box). The bottom graphene sheet in the middle part is characterized by a curvature of 0.173 nm^−1^ and a radius of curvature of 5.754 nm. The upper graphene sheet is characterized by a curvature of −0.161 nm^−1^ and a radius of curvature of −6.235 nm. Therewith, the curvature changes along the direction of *y*-axis; in the direction of *x*-axis, it remains constant at each point of the graphene sheet. The packing density of DPPC molecules in this case is one molecule at 4.4 nm^2^, which is 33% higher as compared to the original atomistic model of bilayer phospholipid-graphene film in Figure 3b. Figure 4b shows how the potential energy of phospholipid-graphene bilayer film changed during the search for the equilibrium configuration of the film supercell in the first picosecond of MD simulation. The energy of the film in the equilibrium configuration *E_equ_* is −19,224.193 eV, the average deviation of the potential energy *E_pot_* from the equilibrium value was 0.012 eV. The equilibrium configuration of the film was achieved after ~2 ps. The behavior of the RMSD clearly shows that the atomic structure of a phospholipid-graphene bilayer film tends to an equilibrium state by the end of the first picosecond of MD simulation.

**Figure 5:**
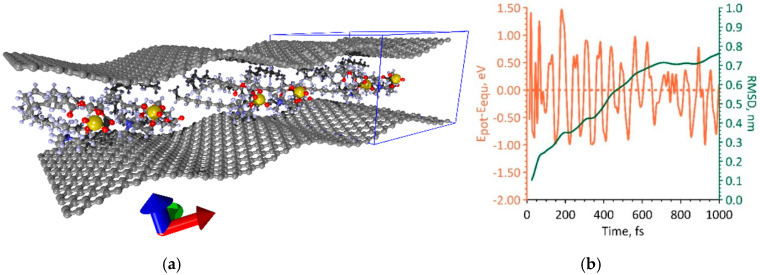
The second variant of dense packing of DPPC molecules in bilayer phospholipid-graphene film: (**a**) atomistic model of bilayer phospholipid-graphene film; and (**b**) changes in potential energy *E_pot_* relative to its equilibrium value *E_equ_* and RMSD during the formation of an equilibrium configuration of film.

During the simulation of the second way of increasing the packing density of DPPC molecules, it was shown that a supercell of a bilayer phospholipid-graphene film with dimensions of 26.50 × 29.78 Å is energetically favorable. This supercell contains two DPPC molecules interacting with each other, as shown in Figure 5a. The graphene layer is formed by 308 atoms. In this case, a wave-like bending of curved graphene is observed along the *x*-axis and not the *y*-axis, as was the case in the first variant of increasing the packing density. Indeed, the curvature of graphene varies only along the *x*-axis. The maximum curvature of graphene is 0.364 nm^−1^ and the radius of curvature is 2.741 nm. Both graphene sheets have almost the same curvature. The value of this curvature exceeds 2.1 times the value of the graphene sheet curvature in the first variant of packing of DPPC molecules (Figure 4a). The packing density of DPPC molecules in this case is one molecule per 3.9 nm^2^, which is 11% higher as compared to the previous variant. Figure 5b shows a graph of the change in the potential energy during the search for the equilibrium configuration of the film supercell in the first picosecond of MD simulation. The value of the equilibrium energy *E_equ_* = −35,833.262 eV, and the average deviation of the potential energy from the equilibrium value was 0.21 eV. The equilibrium configuration of a bilayer phospholipid-graphene film was reached after ~2.5 ps; however, the RMSD value reached a plateau already at the time points of 750–800 ps and then changed slightly.

**Figure 6:**
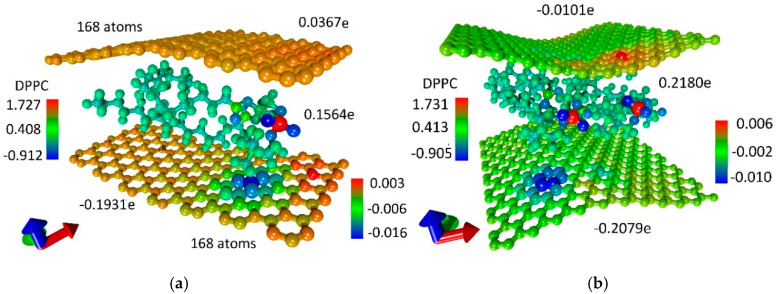
Map of the electron charge density distribution over atoms of a bilayer phospholipid-graphene film: (**a**) the first atomistic model with one DPPC molecule in a supercell; and (**b**) the second atomistic model with two DPPC molecules in a supercell.

For both constructed atomistic models of a bilayer phospholipid-graphene film with dense packing of DPPC molecules, the distribution of the electron charge density over atoms was calculated. The calculation results are presented in Figure 6. This figure demonstrates two supercell atomistic models and the electron charge distribution shown in color. The difference between two atomistic models is noticeable, primarily in the amount of charge transferred onto the graphene sheets. In the case of the first atomistic model, the upper graphene sheet has a small positive charge while in the case of the second atomistic model a slight negative charge. The negative charge on the bottom graphene sheet of the second atomistic model is greater by ~8% as compared with the first atomistic model. In general, the effect of increasing charge transfer to graphene has been achieved. As a result of the increase in the packing density of DPPC molecules, the bottom graphene layer of the first atomistic model of a phospholipid-graphene bilayer film took up 49.8% more charge in terms of one graphene atom in the supercell as compared with the initial atomistic model of a phospholipid-graphene bilayer film shown in Figure 2b. On the contrary, the bottom graphene layer in the second atomistic model of phospholipid-graphene bilayer film took up 11.9% less charge in terms of one graphene atom in the supercell as compared with the initial atomistic model of a phospholipid-graphene bilayer film shown in Figure 2b. However, in general, the charge on graphene in the supercell of a bilayer phospholipid-graphene film increased, which should also lead to an increase in electrical conductivity of this film. The pattern of the negative charge distribution over graphene in the second atomistic model of phospholipid-graphene film (Figure 6b) allows us to conclude that all graphene atoms have a negative charge in the range (−0.01; −0.001)*e*, which also plays a positive role in the current transfer. In the first model, on the contrary, graphene atoms with a lack of electron charge are observed; they can be clearly seen in Figure 6a. The positive charge for some atoms is ~0.002*e*.

### 3.4. The Electrical Conductivity of Graphene Layers in Bilayer Phospholipid-Graphene Films with Curved Graphene and the Effect of Curvature on the Sensory Ability of Films

**Figure 7:**
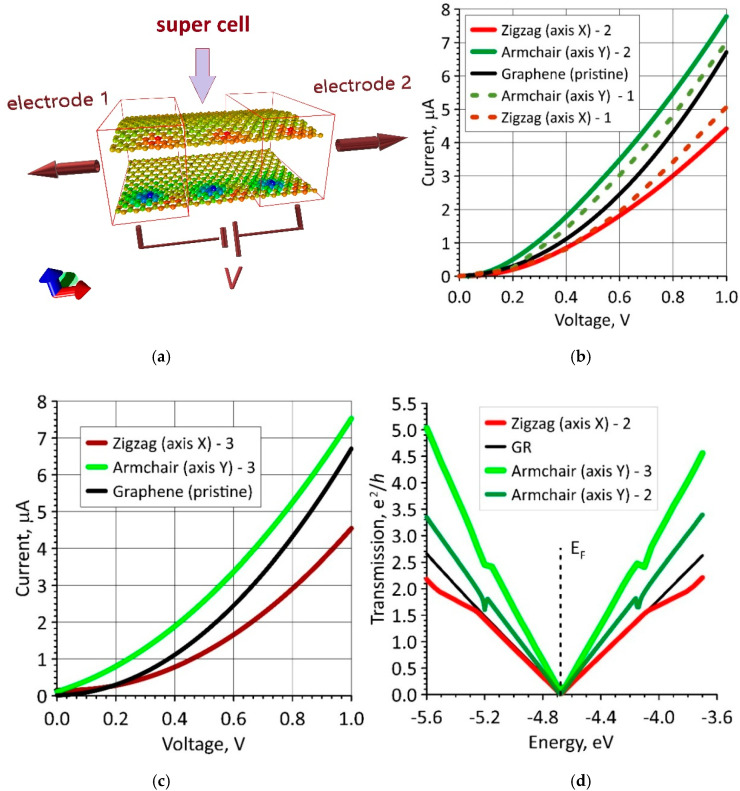
Current-voltage (*I*–*V*) characteristics of bilayer phospholipid-graphene bilayer films: (**a**) scheme for connecting graphene layers with a transferred charge to the supercell electrodes; (**b**) *I*–*V* characteristics for two models with one DPPC molecule in a supercell (the number “1” corresponds to a supercell of 22.14 × 29.82 Å in size, the number “2” corresponds to a supercell of 14.76 × 29.81 Å in size); (**c**) *I*–*V* characteristics for model, where the supercell contains two DPPC molecules (indicated by the number “3”); and (**d**) transmission functions for Models “2” and “3” of a phospholipid-graphene film (black curves correspond to graphene).

To clarify and better understand the effect of the curvature and packing density of DPPC molecules on electrical conductivity and sensory properties of bilayer phospholipid-graphene films, we calculated the current–voltage (*I*–*V*) characteristics of graphene layers in phospholipid-graphene films taking into account the calculated redistribution of electron charge density in the films. To simulate the current transfer through a given supercell in a given direction, for example, along the *x*-axis, an adjacent supercell in the negative direction of the *x*-axis is selected as electrode 1 and it is translated to infinity in this direction. A neighboring supercell in the positive direction of the *x*-axis is selected as electrode 2, and it is translated to infinity in this direction. Some voltage *V* is applied between the electrodes; its value varies in the interval (0–1) *V* with a step of 0.01 V. Such a connection to the electrodes is shown in Figure 7a. Herewith, all three supercells are translated to infinity in direction of the *y*-axis perpendicular to the direction of current transfer. DPPC molecules are not shown in Figure 7a, since they do not connect to the electrodes and do not participate in current transfer. The color map in Figure 7a displays the distribution of the electron charge density and corresponds to the atomistic model of a bilayer phospholipid-graphene film shown in Figure 6a.

The *I*–*V* characteristics were calculated for two atomistic models of a phospholipid-graphene film with one DPPC molecule in a supercell: (1) a model with a supercell of 22.14 × 29.82 Å in size containing 504 carbon atoms; and (2) a model with a supercell of 14.76 × 29.81 Å in size containing 336 carbon atoms. These atomistic models of the film differ slightly in curvature, but they differ significantly in charge transfer. Figure 7b shows the *I*–*V* characteristics for a planar individual graphene monolayer (black curve): two dashed *I*–*V* curves for the model with a supercell of 22.14 × 29.82 Å in size (they are indicated by the number “1”) and two solid I–V curves for the model with a supercell of 14.76 × 29.81 Å in size (they are indicated by the number “2”). The *I*–*V* curves for current transfer in the zigzag direction of graphene are marked in red, and the *I*–*V* curves for current transfer in the armchair direction are marked in green. Both directions of current transfer are the same for graphene, but they differ markedly for phospholipid-graphene films. Both atomistic models of the films are characterized by a rapid increase in current in the armchair direction with increasing voltage. For example, at a voltage of 0.4 V, the difference between the current values in the zigzag and armchair directions is 87.5% and 125%, respectively. A similar noticeable domination of the armchair direction in current transfer is also observed in the third atomistic model of the film with two DPPC molecules in one supercell. The *I*–*V* curves of this atomistic model are shown in Figure 7c (they are indicated by the number “3”), as well as the *I*–*V* curves of graphene for comparison. In this case, at a voltage of 0.4 V, the current value is insignificant, but greater than for atomistic models with one DPPC molecule in a supercell. At a voltage of 0.2 V, the current is ~30% greater (comparison of the green solid curves in Figure 7b,c). That is, the maximum rapid increase in current at low voltages of 0–0.2 V is observed in the armchair direction and only for bilayer phospholipid-graphene films with two DPPC molecules in one supercell (the supercell is shown in Figure 5a). As is known, the rapid current response to low voltages indicates a high sensory ability of this material.

To clarify the reasons for this difference in the *I*–*V* characteristics for indicated three atomistic models of a bilayer phospholipid-graphene bilayer film, we calculated the transmission functions *T*(*E*) for each of them. The calculated *T*(*E*) functions for Models “2” and “3” are presented in Figure 7d. The colors of the *T*(*E*) curves meet the colors of the corresponding *I*–*V* curves for these models in Figure 7b,c. The bright green *T*(*E*) curve stands out in Figure 7d. This curve determines the *I*–*V* characteristic for an atomistic model with two DPPC molecules in the armchair direction of current transfer. It can be seen that the function *T*(*E*) in this case sharply increases near the Fermi level (the dashed vertical line) in comparison with all other cases. This explains the rapid increase in current at low voltages.

## 4. Discussion and Conclusions

Monolayer and bilayer phospholipid-graphene films were studied from the standpoint of improving their sensory properties. One of the important points for use of material in sensors is the rapid response of the material, that is, the appearance of an electric current in the circuit even at low voltages. We found the optimal configuration of the arrangement of DPPC molecules between two graphene layers, which provides the best *I*–*V* characteristics. As can be seen in Figure 7, at a voltage of 0.1 V, the current is 0.4 μA, and, at a voltage of 0.24 V, the current is already 1 μA. This effect is ensured by charge transfer from DPPC molecules to one of the graphene layers. The charge transfer provides a change in the electron transmission function *T*(*E*) as compared to the initial graphene, providing a sharp increase in the electron conductivity near the Fermi level. Note that the optimal configuration of the arrangement of DPPC molecules, which provides the above effects, is achieved only in the case of curved graphene. The maximum effect of charge transfer is observed when the radius of curvature becomes equal to ~2.7 nm at some points of graphene layers.

Thus, bilayer phospholipid-graphene films can be used in sensors, with one of the graphene layers acting as the working surface. One of graphene layers has a negative charge of ~(−0.2e) and the other graphene layer is almost neutral. It also favorably distinguishes such bilayer films in comparison with monolayer ones, since the charged graphene layer will more actively manifest itself due to the electrostatic effect.
